# Evaluating the Success of Immediate Implants in the Esthetic Zone: A Narrative Review with Case Illustration

**DOI:** 10.3390/dj13080365

**Published:** 2025-08-12

**Authors:** Carlos A. Jurado, Francisco Garcia-Torres, Silvia Rojas-Rueda, Kiarash Karimi, Mark Adam Antal

**Affiliations:** 1 Division of Operative Dentistry, Department of General Dentistry, The University of Tennessee Health Science Center College of Dentistry, Memphis, TN 38104, USA; 2 School of Dentistry, Ponce Health Sciences University, Ponce 00716, Puerto Rico; 3 Department of Prosthodontics and Implantology, School of Dentistry, University of La Salle, Leon 37150, Mexico; 4 Division of Dental Biomaterials, The University of Alabama at Birmingham School of Dentistry, Birmingham, AL 35233, USA; 5 Department of Restorative Sciences, School of Dentistry, University of California, Los Angeles, CA 90024, USA; 6 Department of Operative and Esthetic Dentistry, Faculty of Dentistry, University of Szeged, 6720 Szeged, Hungary

**Keywords:** immediate implant, esthetic zone, ceramics, veneers, immediate loading, implant dentistry

## Abstract

**Background**: Immediate implant therapy is a highly effective solution for replacing non-restorable teeth, particularly in the esthetic zone, where achieving optimal results can be challenging. In this area, even small imperfections can significantly affect a patient’s satisfaction due to the high visibility of the teeth involved. This narrative review provides an overview of findings from case reports and systematic reviews that highlight the success of immediate implant therapy in the esthetic zone. Additionally, it includes a case illustration to emphasize how meticulous planning, combined with advanced techniques, can achieve successful outcomes. **Methods**: A comprehensive literature review was conducted to evaluate the effectiveness of immediate implant placement and loading for non-restorable teeth in the esthetic zone. Key factors identified for success include atraumatic tooth extraction, precise implant placement, and effective soft tissue management to achieve natural esthetics and long-term stability. To illustrate these principles, the review features a clinical case involving the replacement of a maxillary right central incisor rendered non-restorable due to trauma. Treatment incorporated advanced digital planning, atraumatic extraction, immediate implant placement, and provisionalization. The final restoration involved soft tissue contouring and ceramic veneers on adjacent teeth, enhancing the patient’s overall smile and confidence. **Results**: Evidence from the literature indicates that well-planned immediate implant therapy achieves high success rates and long-term stability. In the clinical case presented, the workflow met the patient’s esthetic and functional needs, resulting in a natural, harmonious smile, and improved patient satisfaction. **Conclusions**: Immediate implant therapy in the esthetic zone is highly effective when critical considerations—such as bone preservation, guided implant placement, soft tissue shaping, and appropriate restoration—are meticulously addressed. Advanced techniques and careful planning are essential for fulfilling both esthetic and functional patient needs, ultimately delivering predictable and successful outcomes.

## 1. Introduction

In the esthetic zone, the replacement of missing teeth necessitates a comprehensive evaluation of various prosthetic options to achieve optimal functional and esthetic outcomes. Each option comes with distinct advantages and limitations, which must be carefully weighed. Fixed dental prostheses (FDPs), a traditional solution, provide immediate restoration by anchoring to adjacent teeth. However, they require the preparation of healthy abutment teeth, a consideration that may not align with patient preferences or clinical circumstances [1]. Despite this, FDPs remain a reliable choice for restoring function and esthetics. Removable partial dentures (RPDs) offer a minimally invasive alternative and are particularly suitable for patients with significant tooth loss or those seeking a cost-effective option. Modern advancements, such as rotational path RPDs, have improved the esthetic appeal and comfort of these prostheses, making them a viable choice for many individuals [2]. Dental implants, on the other hand, are increasingly favored for their ability to preserve adjacent dental structures and provide a stable, esthetically pleasing solution. The high survival rates of dental implants, ranging from 96.4% to 100%, underscore their reliability, even when supporting complex restorations like two-unit cantilever prostheses in esthetically sensitive areas. Furthermore, patients report high satisfaction with dental implants, citing their stability and natural appearance as key benefits [3]. Ultimately, the selection of a prosthetic option should be individualized, taking into account the patient’s oral health status, anatomical considerations, esthetic expectations, and financial resources. By tailoring the treatment plan to these factors, clinicians can ensure an outcome that aligns with the patient’s needs and preferences [4].

Dental implants have evolved significantly over centuries, tracing their roots back to ancient civilizations. As early as 600 AD, the Mayans demonstrated remarkable ingenuity by implanting carved seashells into their mandibles. Fascinatingly, some of these primitive implants exhibited integration with the surrounding bone tissue, an early precursor to the concept of osseointegration [5]. The field began taking a more scientific turn in 1913 with the introduction of the Greenfield crib implant. Constructed from iridioplatinum, this implant was a significant advancement and provided early evidence of osseointegration, although the full potential of this phenomenon was not yet understood [6]. During the 1940s and 1950s, researchers Bothe, Beaton, and Davenport laid the foundation for modern implantology by identifying bone’s natural affinity for titanium. Their findings sparked further exploration into the relationship between bone and implant materials, paving the way for significant breakthroughs. A transformative milestone occurred in 1965 when Dr. Per-Ingvar Brånemark successfully placed a titanium dental implant in a human patient. This achievement not only confirmed titanium’s biocompatibility but also established the concept of osseointegration as a cornerstone of implant dentistry. Brånemark’s pioneering work laid the groundwork for contemporary practices and opened the door to innovations that continue to shape the field [7]. Since that time, dental implantology has advanced rapidly, driven by innovations in digital technology and materials science. Digital imaging and computer-aided design/manufacturing (CAD/CAM) have improved precision in implant placement, while new materials have enhanced durability, functionality, and esthetics. Today, dental implants are recognized as a reliable and highly effective solution for replacing missing teeth, offering patients improved oral function and esthetic outcomes [8].

Implant therapy offers a host of benefits that set it apart from traditional options such as fixed bridges and removable partial dentures, making it an increasingly favored choice for tooth replacement. Unlike bridges, dental implants do not require the preparation or alteration of adjacent teeth. By preserving the natural structure of surrounding teeth, implants help to avoid potential long-term complications, such as weakening or decay of the abutment teeth, which can compromise the overall integrity of the dentition [9,10]. In contrast, removable partial dentures (RPDs) often lead to patient dissatisfaction due to their bulkiness, discomfort, and the esthetic compromises they impose. These drawbacks can significantly affect a patient’s quality of life, particularly in cases where esthetics are a primary concern. Dental implants, however, offer superior functional outcomes, including enhanced masticatory efficiency and improved comfort, which make them especially suitable for replacing teeth in the anterior zone, where esthetic demands are highest [11,12]. Another key advantage of dental implants is their ability to maintain alveolar bone and counteract bone resorption. This critical benefit is not achievable with conventional bridges or dentures, which do not stimulate the underlying bone in the same way. Over time, this preservation of bone structure contributes to better oral health and improved facial esthetics [13]. Additionally, dental implants are recognized for their durability and reliability. Longitudinal studies consistently report high survival rates, ranging from 95.2% to 97.5% over a 10-year period. These impressive figures highlight the long-term effectiveness of implant therapy, making it a dependable solution for patients seeking stable, natural-looking, and functional tooth replacement [14].

Implant loading protocols are categorized into immediate, early, or delayed loading, depending on the timing of prosthesis placement relative to the placement of the dental implant [15]. These protocols aim to optimize both the functional and esthetic outcomes of implant therapy while ensuring implant stability and long-term success. Immediate loading involves placing the dental implant into the extraction socket and attaching the prosthesis on the same day as the tooth extraction. This approach offers the advantage of reducing treatment time and providing immediate esthetic and functional restoration. Studies have reported survival rates of approximately 95–98% over several years of follow-up, making it a reliable option for carefully selected cases with good bone quality and primary stability [16,17,18]. Early loading represents a middle ground, where implants are restored after a period of soft tissue healing (4–8 weeks) or partial bone healing (12–16 weeks) following tooth extraction. This protocol allows for some initial bone remodeling and stabilization while offering a faster restoration timeline compared to delayed loading. It is particularly beneficial for patients who prioritize reduced waiting times without compromising implant integration [19]. Delayed loading, in contrast, remains the most predictable and widely used protocol, especially in cases of compromised bone quality or when osseointegration is uncertain. This approach involves waiting for complete bone healing, typically more than six months after tooth extraction, before placing the prosthesis. By allowing sufficient time for bone maturation and integration, delayed loading minimizes the risk of implant failure and is considered the gold standard in challenging clinical scenarios [20]. Each loading protocol has its specific indications and considerations, and the choice should be tailored to the patient’s clinical condition, anatomical factors, and treatment goals. By selecting the appropriate protocol, clinicians can optimize both short- and long-term outcomes for implant-supported restorations.

Immediate loading offers numerous advantages, making it an attractive option for both patients and clinicians in carefully selected cases. One of the most significant benefits is the reduction in overall treatment time, which enhances patient comfort and satisfaction. By minimizing the duration of the treatment process, patients experience less disruption to their daily lives and enjoy quicker functional and esthetic restoration [21].

This technique also helps preserve critical anatomical and cosmetic features by reducing bone resorption, maintaining natural gum contours, and supporting the alveolar bone and surrounding soft tissues. These factors are essential for achieving optimal esthetic outcomes, particularly in the anterior region where appearance is a primary concern [21]. Immediate implants streamline the overall treatment process, as they typically require fewer surgical procedures. This leads to less cumulative trauma, reduced time spent in the dental office, and a quicker return to normal function. For many patients, the placement of a temporary crown during the same visit allows them to benefit from immediate esthetic improvement and a significant boost in self-confidence [22,23]. Moreover, the immediate placement of implants prevents the movement of adjacent teeth, helping to maintain proper tooth alignment and ensuring ideal positioning and functionality of the final restoration. This benefit simplifies future prosthetic work and contributes to long-term treatment success. These combined advantages make immediate dental implants an efficient, cost-effective, and esthetically pleasing solution for replacing missing teeth in suitable cases. However, careful case selection remains critical, as the success of this approach relies on factors such as sufficient bone quality, primary implant stability, and overall oral health [24].

The purpose of this narrative review is to evaluate the clinical predictability and success of immediate implant placement and loading in the esthetic zone, focusing on findings from case reports and systematic reviews available in the literature. This review highlights essential biological, surgical, and prosthetic factors that contribute to the success of immediate implant therapy, emphasizing the importance of meticulous planning and execution. Key considerations include achieving optimal esthetic outcomes, effective soft tissue management, and ensuring long-term stability in appropriately selected patients. The discussion underscores how these factors interplay to influence the success of immediate implant placement in a highly visible area where even minor imperfections can impact patient satisfaction.

To complement the findings from the literature, a representative clinical case is presented, illustrating a step-by-step approach to the planning and execution of immediate implant therapy. The case involves a patient with a non-restorable maxillary central incisor due to trauma. The workflow included digital planning, atraumatic extraction, immediate implant placement with a surgical guide, and provisionalization. Final treatment steps involved contouring the soft tissue and placing a definitive implant-supported crown, along with veneers, on adjacent teeth to enhance the overall esthetic outcome. This case demonstrates how immediate implant therapy, when carefully planned and executed, can successfully meet a patient’s esthetic and functional demands while delivering predictable and durable results.

## 2. Materials and Methods

### 2.1. Literature Review

A comprehensive literature search was conducted to identify manuscripts published between January 2010 and March 2025 focusing on immediate implant therapy in the esthetic zone. The search was performed using PubMed, Scopus, Web of Science, and Google Scholar, employing the following search strategy: (“Immediate Implant in the Esthetic Zone”), (“Immediate Implant in the Anterior Area”) AND (“Immediate Implant in the Esthetic Zone Review”). The initial search yielded a total of 328 articles. Titles and abstracts were screened, and 20 manuscripts were selected for detailed review. These included 10 case studies and 10 reviews, encompassing systematic reviews and meta-analyses. Several exclusion criteria were applied to refine the selection process. Publications dated prior to January 2010, non-English language articles, letters, books, book chapters, and manuscripts without full-text availability were excluded. The inclusion and exclusion criteria are detailed in Table 1. This selection process ensured the inclusion of relevant and high-quality studies, facilitating a focused analysis of the outcomes and considerations for immediate implant therapy in the esthetic zone. The chosen studies provide insights into clinical outcomes, patient satisfaction, and procedural advancements within this specialized field of implant dentistry.

### 2.2. Case Report

**Figure 1:**
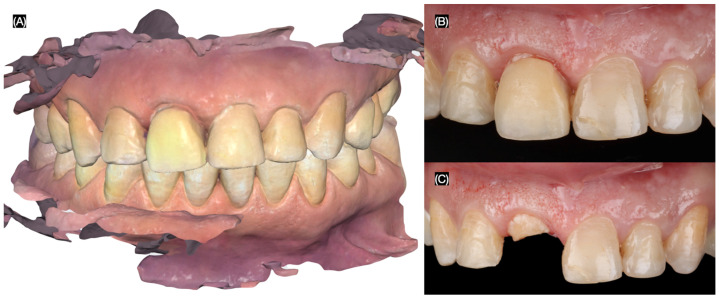
Initial situation. (**A**) Intra-oral scan of maxilla and mandible in occlusion. Maxillary anterior teeth (**B**) with and (**C**) without crown on right central incisor.

A 32-year-old female patient presented with the chief complaint of mobility in a front crown. She reported playing basketball and experiencing trauma to her mouth after being struck by the ball. Additionally, the patient expressed a desire to improve her overall smile esthetics. To begin the diagnostic process, intra-oral photos were taken, and an intra-oral scan (Medit i600, Seoul, South Korea) was performed for the maxillary and mandibular arches, including an occlusal registration to capture the bite relationship (Figure 1).

**Figure 2:**
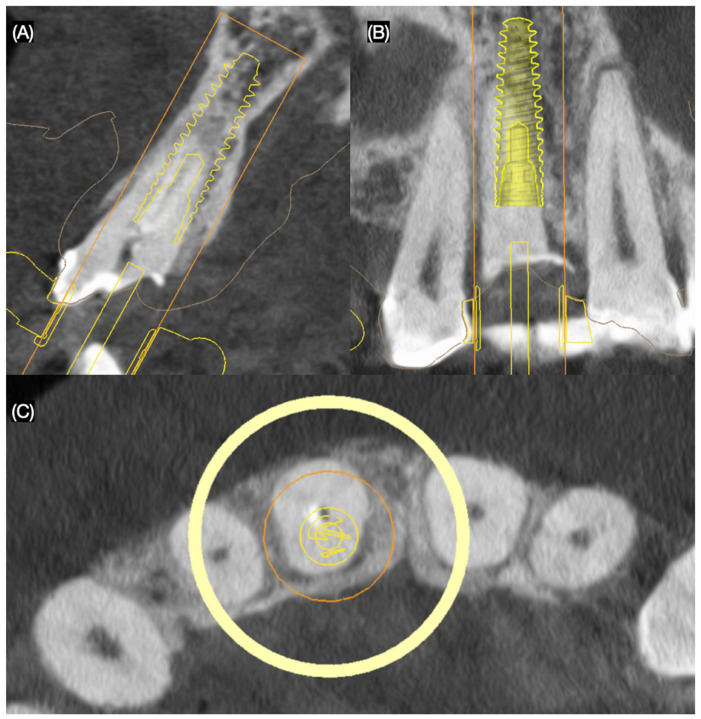
Three-dimensional implant evaluation with CBCT: (**A**) axial, (**B**) frontal, and (**C**) incisal view.

Based on clinical and radiographic findings, the patient was diagnosed with the following: A non-restorable, fractured maxillary right central incisor; old and stained resin composite restorations on both maxillary lateral incisors; and incisal wear on the maxillary canines, lateral incisors, and left central incisor. The patient was informed that due to the extent and location of the fracture, the maxillary right central incisor could not be salvaged and required extraction. A comprehensive treatment plan was proposed to address the patient’s functional and esthetic concerns, including the replacement of the non-restorable tooth and the enhancement of the anterior dentition. The plan consisted of the placement of a single immediate implant to replace the fractured maxillary right central incisor and ceramic veneers for the maxillary right lateral incisor, left lateral incisor, right canine, and left canine. A three-dimensional cone-beam computed tomography implant evaluation was also conducted (MSOFT, MIS Implant, Misgav, Israel) to assess the structural condition of the affected tooth and surrounding tissues. The evaluation revealed a subgingival horizontal fracture of the maxillary right central incisor (Figure 2).

**Figure 3:**
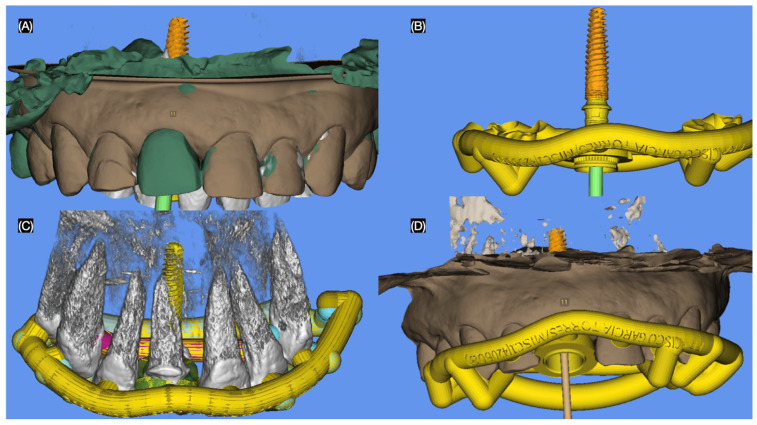
Digital planning of the surgical guide. (**A**) Implant position frontal view, (**B**) guide and implant, (**C**) guide and implant over teeth, and (**D**) guide and implant access frontal view.

Considering the patient’s financial constraints and preferences, she opted for ceramic veneers for the maxillary lateral incisors and a resin composite veneer solution for both canines. To visualize the proposed outcomes, a diagnostic printed (Anycubic Resin 3D Printer Mono 4K, Anycubic, Shenzhen, China) was fabricated based on the patient’s intraoral scan. A traditional wax-up (Anycubic Clear UV Resin, Anycubic, Shenzhen, China) was created on the printed diagnostic model to refine and communicate the desired anatomy of the anterior dentition. The goal of digitally planning the immediate implant placement was to position it 3 mm apical to the crestal bone to achieve initial stability, as recommended in the literature [25,26]. Additionally, a three-dimensional evaluation was conducted to ensure precise treatment planning, and a surgical guide was designed (MSOFT, MIS Implant, Misgav, Israel) to facilitate accurate implant placement (Figure 3).

**Figure 4:**
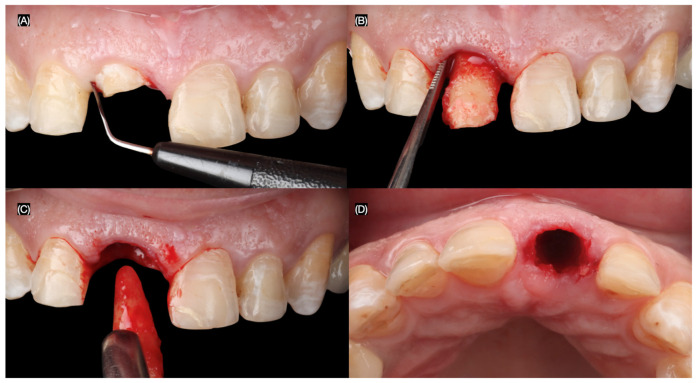
Flapless atraumatic tooth extraction. (**A**) Desmotomes insertion, (**B**) luxation of root, (**C**) extraction, and (**D**) incisal view after extraction.

**Figure 5:**
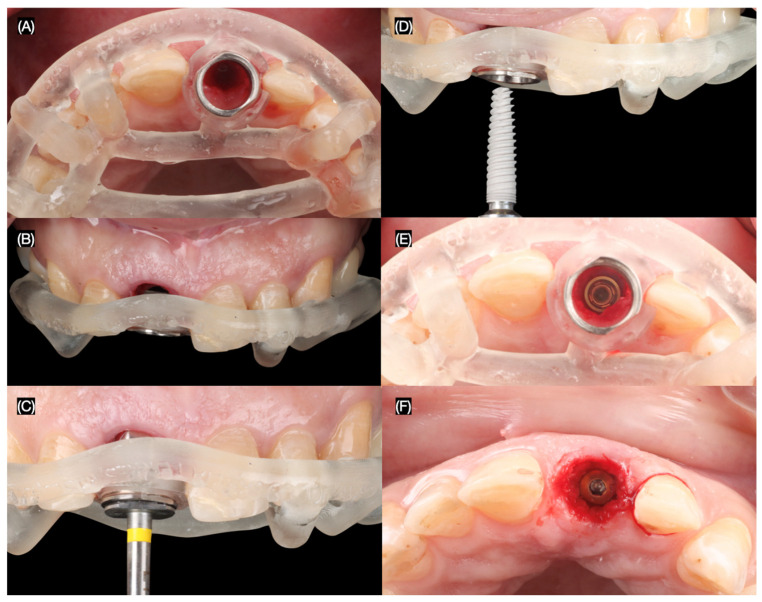
Implant placement. Implant surgical guide: (**A**) incisal and (**B**) frontal view, (**C**) initial pilot drilling, (**D**) implant placement, and implant placed (**E**) with and (**F**) without guide incisal view.

The maxillary right central incisor was extracted atraumatically, preserving the surrounding bone and soft tissues. An immediate implant (MISC C1, 3.30 mm × 13 mm, MIS Implant, Misgav, Israel) was placed using a surgical guide, ensuring precise positioning and primary stability (Figure 4 and Figure 5).

**Figure 6:**
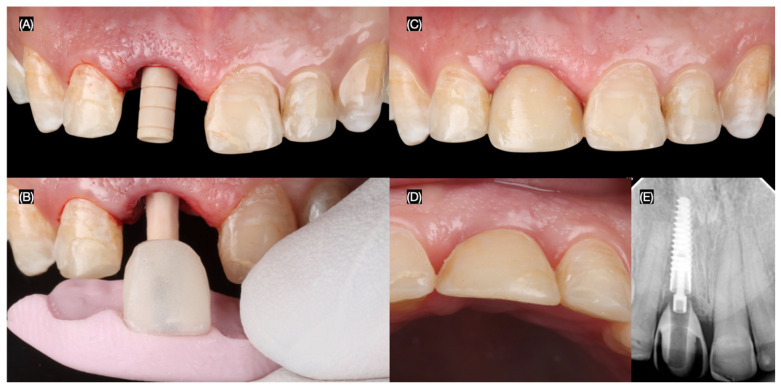
Implant provisional restoration. (**A**) Interim implant post; (**B**) placement of the interim crown with putty index, interim crown, (**C**) frontal, and (**D**) incisal view; and (**E**) radiograph.

Following the implant placement, a screw-retained provisional restoration (Structure Premium, VOCO GmbH, Cuxhaven, Germany) was immediately placed. The provisional restoration was carefully adjusted to be out of occlusion to minimize micromovements that could compromise osseointegration and potentially lead to implant failure (Figure 6).

**Figure 7:**
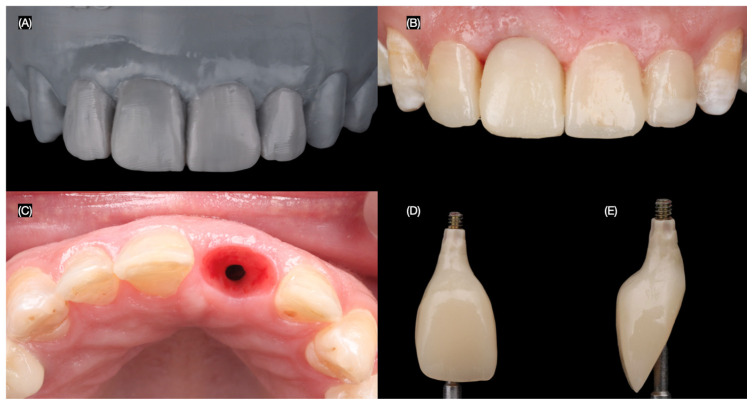
Design of the final restorations. (**A**) Diagnostic wax-up, (**B**) new interim restoration, (**C**) soft tissue contoured, and (**D**) facial and (**E**) proximal view of interim restoration.

To optimize the esthetic outcome, the provisional restoration was recontoured to guide the development of the desired gingival architecture and soft tissue healing. Minimally invasive veneer preparations were then performed on the adjacent teeth using tooth reduction guides (Elite P&P, Zhermack, Badia Polesine, Italy) created from the diagnostic wax-up. These guides ensured precise and conservative tooth reduction to achieve optimal veneer fit and esthetics. A final impression of the implant and prepared teeth was made to fabricate the definitive restorations (Figure 7).

**Figure 8:**
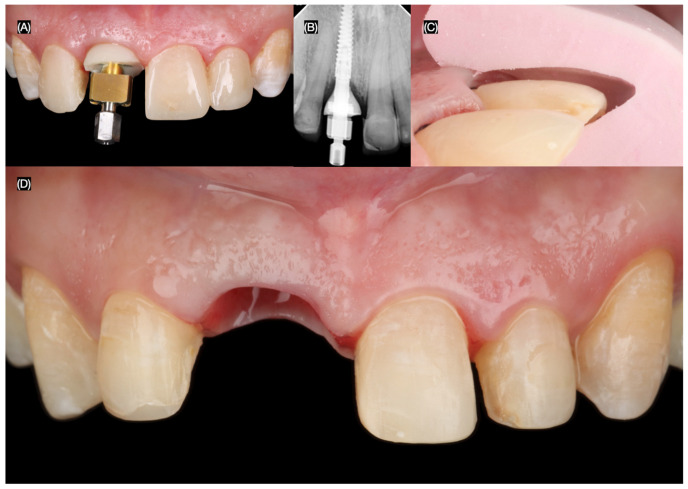
Final tooth reparations and impression. (**A**) Implant impression, (**B**) radiograph with impression post, (**C**) tooth preparation with reduction guide, and (**D**) frontal view of contoured soft tissue and tooth preparations.

**Figure 9:**
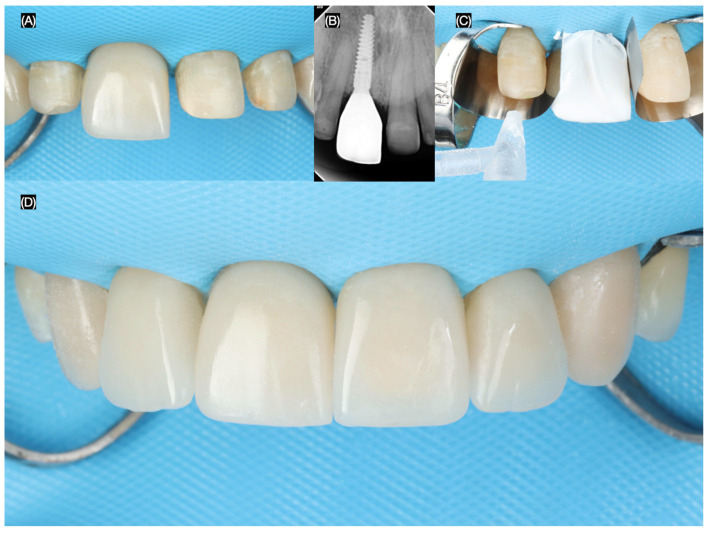
Placement of the final restorations under dental dam isolation. (**A**) Implant restoration, (**B**) radiograph of implant restoration, (**C**) tooth surface treatment with sandblast, and (**D**) final cementation of restorations.

The final screw-retained implant crown was placed, ensuring proper fit, occlusion, and esthetics. A dental dam was then applied to achieve total isolation for the cementation of the veneers, ensuring a clean and controlled environment. The tooth surfaces were first prepared using sandblasting, followed by etching with phosphoric acid. The lithium disilicate veneers were treated with hydrofluoric (Porcelain Etchant, Bisco, Schaumberg, IL, USA) acid for 20 s, thoroughly cleaned in an ultrasonic water bath for 5 min, and coated with a silane (Porcelain Primer, Bisco, Schaumberg, IL, USA) coupling agent for 60 s. Finally, the veneers were bonded using universal adhesive (All Bond Universal, Bisco, Schaumberg, IL, USA) and dual-cure resin cement (Choice 2 Veneer Cement, Bisco, Schaumberg, IL, USA), ensuring a durable and esthetic outcome (Figure 8 and Figure 9).

**Figure 10:**
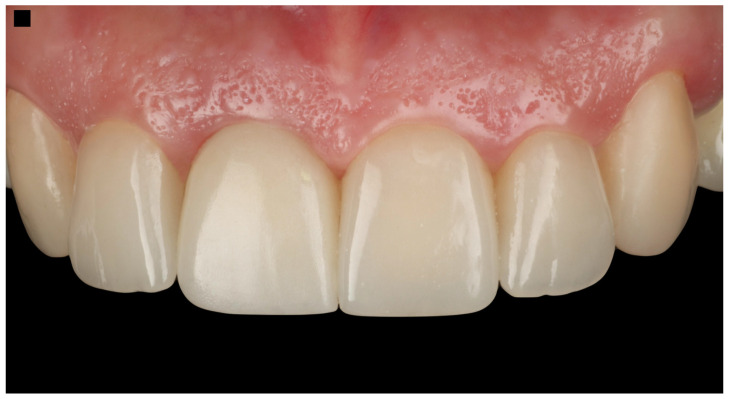
Final restorations.

The patient expressed complete satisfaction with the final restorations, praising the natural shade and shape of the teeth. To protect the restorations from potential damage, the patient was provided with a custom-made night guard. Detailed oral hygiene instructions were given, emphasizing the importance of maintaining the health of the restorations and surrounding tissues. The patient was scheduled for regular follow-up visits every six months for professional dental prophylaxis and to monitor the condition of the restorations and periodontal tissues (Figure 10). At the three-year follow-up visit, the patient was still satisfied with the outcome.

## 3. Results

### 3.1. Results of the Literature Review

To provide a comprehensive understanding of the reviewed information, the articles were systematically categorized into two distinct tables. This approach aims to enhance clarity and facilitate comparison of findings across studies. The first table, Table 2 [27,28,29,30,31,32,33,34,35,36], focuses on case reports detailing clinical protocols and outcomes for immediate implant therapy performed in the esthetic zone. These reports highlight various clinical techniques, patient-specific considerations, and follow-up outcomes, offering valuable insights into the real-world application of immediate implant therapy. The data include aspects such as patient demographics, implant placement protocols, soft tissue management, prosthetic restoration techniques, and clinical outcomes, such as esthetics, implant survival rates, and complications. The second table, Table 3 [37,38,39,40,41,42,43,44,45,46], summarizes systematic reviews that critically evaluate the effectiveness of immediate implant therapy in the esthetic zone. These reviews consolidate evidence from multiple clinical studies, providing a broader perspective on the efficacy, predictability, and challenges of immediate implant therapy. Key parameters assessed include success rates, esthetic outcomes, peri-implant bone preservation, soft tissue response, patient satisfaction, and potential risk factors affecting treatment outcomes.

Together, these tables aim to bridge the gap between individual case reports and broader systematic analyses, enabling clinicians to make informed decisions when implementing immediate implant therapy in the esthetic zone. This structured presentation underscores the practical applications and evidence-based evaluation of this treatment modality.

### 3.2. Results of the Case Report

The workflow performed in this case illustration successfully addressed the patient’s esthetic and functional needs. The initial three-dimensional planning, including a detailed CBCT analysis, allowed for precise treatment preparation. This was followed by atraumatic tooth extraction and immediate implant placement using a surgical guide. The placement of a screw-retained provisional restoration and the subsequent final restoration were meticulously executed, resulting in a predictable and satisfactory outcome. The restorations on the adjacent teeth significantly enhanced the patient’s smile, improving overall symmetry, color, and shape. Additionally, the patient was provided with a custom-made occlusal guard to protect the restorations during nighttime grinding or clenching.

**Figure 11:**
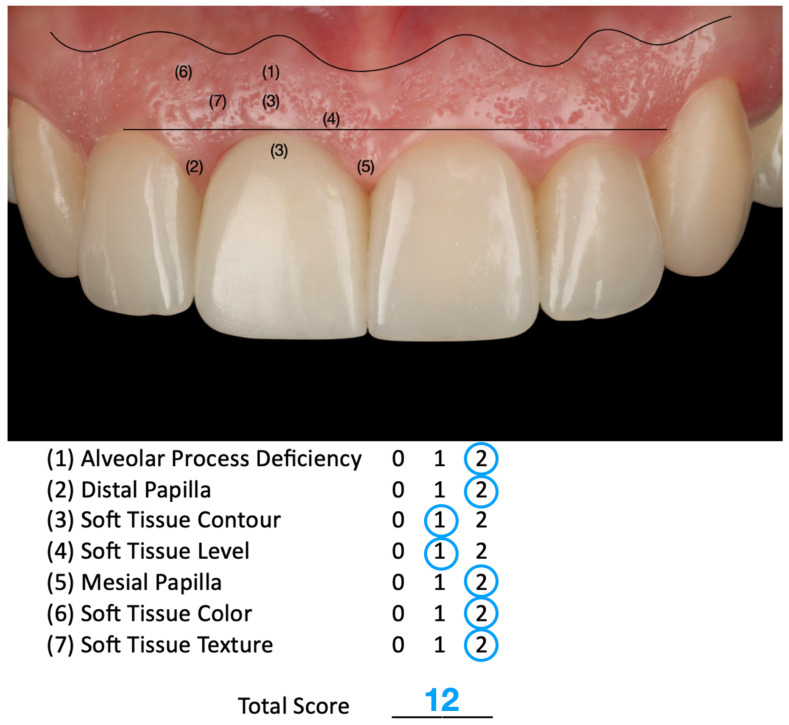
The Pink Esthetic Score (PES) system used to evaluate the outcome of the case illustration.

The Pink Esthetic Score (PES) is a system used to evaluate the esthetic outcomes of soft tissues surrounding dental implants, particularly in the anterior region of the mouth. It is a well-accepted method in the literature for assessing treatment success [47]. The PES evaluates five variables: mesial and distal papilla, curvature of the gingival margin, level of the gingival margin, root convexity, and scar formation. Each variable is scored on a 0–1–2 scale, where 2 represents the best outcome and 0 the poorest. The maximum score is 14, with scores of 11 or above generally considered excellent, scores between 8 and 11 deemed acceptable, and scores below 8 considered poor. Based on the PES system, the results of the case presented in this study provide a score of 12, which is considered an excellent result (Figure 11).

To ensure the long-term success of the treatment, the patient was educated on an oral hygiene protocol. This included detailed instructions on proper brushing and flossing techniques, with particular focus on cleaning around the implant and adjacent restorations. The patient was also placed on a six-month maintenance recall schedule. These visits will include professional dental prophylaxis and a comprehensive evaluation of the restorations, the soft tissues surrounding the implant, and the overall oral health.

**Figure 12:**
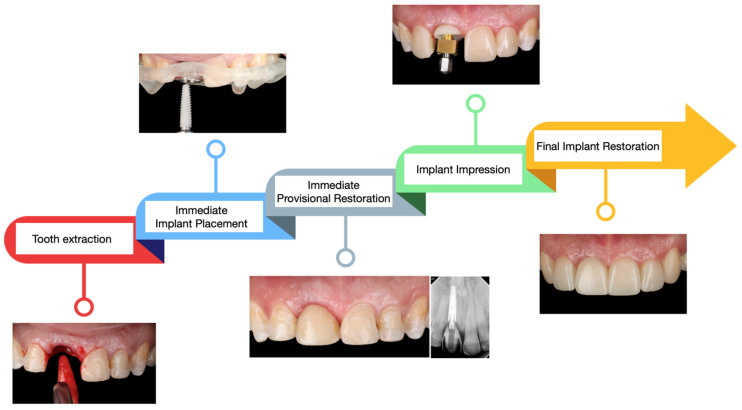
Summary of the clinical steps performed.

The follow-up period for this case illustration is three years. At this stage, the patient remains satisfied with the functional and esthetic outcomes of the implant therapy provided. However, it is important to note that three years is a relatively short period to deem the treatment a long-term success. The patient has been educated on the importance of maintaining good oral hygiene and attending regular follow-up evaluation appointments. The clinical workflow, showcasing the key steps in the treatment process, is summarized in Figure 12. This workflow demonstrates the careful integration of planning, surgical precision, and restorative excellence in achieving the desired patient outcomes.

## 4. Discussion

### 4.1. Challenges of Dental Care in the Smile Zone

Patient esthetic demands have significantly increased over time. Some patients, even after severe traumatic injuries resulting in substantial and irreversible damage, may expect complete recovery and perfect esthetic outcomes [48]. Unfortunately, patients may not fully understand the level of difficulty involved in repairing such trauma, especially when it leads to tooth loss. Therefore, when treating patients with complex conditions, careful assessment is essential to ensure realistic outcomes and avoid failing to meet patient expectations.

The literature has emphasized that immediate implant therapy should not be considered a straightforward procedure. Multiple factors contribute to the complexity of this treatment approach, including the need for atraumatic tooth extraction, proper management of hard and soft tissues, three-dimensional planning, precise implant placement, appropriate selection of implant prosthetic components, soft tissue contouring using provisional restorations, and correct material selection for the final restoration [49,50,51,52].

As a result, in addition to possessing strong clinical skills and conducting thorough diagnosis and treatment planning, the clinician must also educate the patient, assess their expectations, and clearly discuss the desired esthetic outcomes and any limitations of the treatment.

### 4.2. Long-Term Success

Immediate implant therapy has demonstrated impressive long-term success rates in the dental literature, making it a viable and effective treatment option for many patients. Studies consistently report survival rates ranging from 90% to 96% over 3 to 5 years, with some studies documenting rates exceeding 95% at the 10-year mark, reinforcing its durability and reliability [27,28]. Clinical outcomes suggest that immediate implants can deliver highly predictable results, including excellent esthetic outcomes and minimal peri-implant bone loss. These favorable results have been observed even in challenging cases, such as periodontally compromised sites or posterior regions, where bone and tissue conditions are often less than ideal [29]. The ability to achieve stable esthetic and functional results in such situations underscores the versatility and effectiveness of immediate implant placement. Despite these advantages, prosthetic complications such as screw loosening or the loss of proximal contact between adjacent teeth may occasionally occur. However, research has shown that these issues have no significant impact on the overall success of the treatment or on patient satisfaction. This suggests that while prosthetic adjustments may be necessary, they do not compromise the long-term stability or effectiveness of the implant [30]. Comparative studies between immediate and delayed implant placement protocols further validate the efficacy of immediate implants. Research indicates that immediate placement offers bone preservation and esthetic outcomes that are equivalent to, or slightly better than, those achieved with delayed protocols. These findings highlight the importance of proper case selection and adherence to meticulous surgical and prosthetic protocols to maximize success rates [31]. When performed under optimal conditions and with appropriate patient selection, immediate implant therapy provides a dependable, efficient, and esthetically pleasing solution, making it a cornerstone of modern implant dentistry.

### 4.3. Three-Dimensional Planning

Three-dimensional (3D) implant planning using Cone Beam Computed Tomography (CBCT) has become a cornerstone of safe, predictable, and successful dental implant therapy. This advanced imaging technique provides high-resolution, 3D views of the bone, teeth, and critical anatomical structures, enabling clinicians to assess bone volume and density and accurately measure distances to key structures such as nerves or sinuses. By offering a comprehensive understanding of the surgical site, CBCT significantly enhances the precision and safety of implant procedures [32,33]. The accuracy of CBCT imaging is well-supported by research, with studies reporting minimal measurement variations of 0.03 to 0.14 mm during registration. Linear measurement differences in implant site planning across various software platforms range from 0.43 to 0.56 mm, demonstrating the high reliability of these systems [34]. Specialized planning software further refines this process, allowing for detailed simulations of implant position, angle, size, and depth. Research has shown intra-rater differences in implant tip location, entry point, and angulation to be as low as 1.3 ± 0.8 mm, 1.0 ± 0.6 mm, and 4.5° ± 3.1°, respectively. These levels of precision significantly reduce surgical risks, such as damage to surrounding structures, while optimizing implant positioning for long-term success [35]. When coupled with CBCT-guided surgical protocols, immediate implant placement has demonstrated survival rates typically exceeding 94% over five years. This robust evidence highlights the role of CBCT-guided 3D planning in achieving not only accurate implant placement but also favorable long-term clinical outcomes. By ensuring precise implant alignment and reducing potential complications, CBCT-based planning enhances patient safety, improves esthetic results, and contributes to the overall reliability of dental implant therapy [36]. As technology continues to evolve, CBCT imaging and associated planning tools will remain integral to advancing the standards of care in implant dentistry, ensuring that clinicians can deliver highly predictable and successful outcomes tailored to each patient’s unique anatomical and functional needs.

### 4.4. Surgical Guide

A surgical guide is a critical tool in ensuring the precise and reproducible placement of dental implants. By transferring the digital treatment plan directly into the patient’s oral cavity, these guides enable precise control over implant position, orientation, and depth, thereby bridging the gap between preoperative planning and intraoperative execution [37]. Beyond accuracy, surgical guides provide additional benefits, such as protecting vital anatomical structures, including nerves and adjacent teeth, by controlling the drill’s direction. This safeguards patient safety while enhancing procedural efficiency, reducing operative time, and improving patient satisfaction [38,39]. The effectiveness of surgical guides is well-documented in systematic reviews and clinical trials, particularly for immediate implant placement. Studies have shown that guided surgery can achieve a mean linear deviation at the entry point of 1.04 mm and an average angular deviation of 3.90°, underscoring the precision of this approach [40]. Similarly, systematic reviews report a mean linear deviation of 1.07 mm at the entry point and a mean angular deviation of 5.26°, reflecting consistent accuracy across various studies [41]. In terms of clinical outcomes, guided surgery contributes to high implant survival rates. For instance, one review noted survival rates of 97.2% over follow-up periods of 1 to 4 years. Another study focusing on 198 implants placed in the mandible reported a cumulative survival rate of 97.5%, emphasizing the reliability of this method [42,43,44]. The integration of surgical guides into implant dentistry not only enhances precision but also supports predictable, long-term success. By reducing the risks associated with manual implant placement and optimizing the clinical workflow, surgical guides represent a valuable advancement in ensuring the safety, efficiency, and satisfaction of both clinicians and patients. As technology continues to evolve, the role of surgical guides in dental implantology is likely to expand further, solidifying their status as an essential component of modern implant procedures.

### 4.5. Immediate Implant Combined with Bone Grafting

The integration of immediate implant placement with bone grafting has emerged as an effective strategy to significantly reduce post-extraction bone loss and promote peri-implant tissue stability. Recent systematic reviews and clinical trials provide robust evidence supporting this approach. For instance, a 2022 meta-analysis evaluating 604 immediate implants across 15 randomized controlled trials demonstrated that gap grafting between the implant and socket wall reduced horizontal buccal bone resorption by 0.59 mm (a 54% reduction) compared to implants placed without grafting. Additionally, this technique decreased apical migration of the midfacial soft tissue level by 0.58 mm, contributing to enhanced soft tissue stability [45,46]. Moreover, clinical trials have highlighted the efficacy of specific bone substitutes and combinations, such as platelet-rich fibrin matrix paired with demineralized freeze-dried bone allograft. These materials have shown the ability to maintain buccolingual widths with exceptionally minimal bone loss, averaging only 0.10 ± 0.09 mm at six months post-procedure [47]. This preservation of bone structure is vital not only for functional success but also for achieving optimal esthetic outcomes, especially in the anterior zone where esthetics are paramount. The benefits of bone grafting during immediate implant placement are further reinforced by critical reviews, which underscore its role in preserving alveolar ridge volume and minimizing socket remodeling. This approach results in improved integration of the implant with surrounding tissues, ensuring both functional stability and an esthetically pleasing outcome for the patient [53]. Furthermore, a recent critical review highlights that the advantages of grafting the buccal gap are particularly pronounced in the anterior maxilla. This region is characterized by a thin buccal bone plate, often measuring 1 mm or less, which makes it more susceptible to resorption and other complications following immediate implant placement. However, current evidence does not strongly favor one type of bone grafting material over another for this procedure. Despite this lack of definitive guidance, xenogeneic grafts derived from bovine sources remain widely used in clinical practice due to their availability and favorable handling properties. This underscores the need for further research to establish clear recommendations on the optimal grafting materials for immediate implant therapy in such anatomically delicate areas [54].

A clinical study evaluated different types of bone augmentation during immediate implant therapy. In the study, 30 patients with a mean age of 23 years received 30 implants. Half of the patients received freeze-dried bone allograft, while the other half received a modified hydroxyapatite bone graft. The implants were clinically and radiographically evaluated at baseline, 3 months, 6 months, 9 months, and 1 year. The results revealed that bone levels were maintained, and the authors concluded that both graft materials were equally effective [55]. As advancements in biomaterials and surgical techniques continue to refine this process, the combination of immediate implant placement with bone grafting is expected to become a standard of care for ensuring predictable and high-quality outcomes in implant dentistry. This methodology not only addresses the challenges of bone loss but also enhances the long-term success and satisfaction of both clinicians and patients.

### 4.6. Immediate Implant and Provisional Restoration

The placement of direct provisionals over implants, particularly in the esthetic zone, has become a widely accepted and dependable technique, provided primary implant stability is achieved. This approach offers multiple advantages, including reduced overall treatment time, minimized surgical interventions, and enhanced patient satisfaction due to the immediate restoration of both esthetics and function [56]. By allowing provisionalization during surgery, this method provides critical support to peri-implant soft tissues, helping to preserve gingival contours and establish a natural emergence profile—factors that are essential for achieving excellent esthetic outcomes [57]. Clinical studies have demonstrated that implants undergoing immediate provisionalization exhibit high survival rates. For example, trials with a mean follow-up period of 31.2 months have reported survival rates as high as 98.25% in fresh extraction sockets, underscoring the reliability of this approach [21]. However, the success of this protocol hinges on meeting certain clinical prerequisites. Achieving sufficient primary stability, typically characterized by an insertion torque greater than 32 Ncm, is essential. Additionally, careful adjustment of the provisional restoration to prevent occlusal loading during the healing phase is crucial to avoid complications and promote optimal outcomes [58]. When performed meticulously, immediate provisionalization contributes to the creation of an ideal emergence profile and offers strong soft tissue support, ensuring both functional stability and superior esthetic results. This method is particularly advantageous in cases where preserving the natural gingival architecture is a priority, as it reduces the need for additional interventions while fostering long-term patient satisfaction [59]. With continued advancements in implant technology and surgical protocols, immediate provisionalization remains a cornerstone procedure for enhancing treatment efficiency and achieving predictable success in implant dentistry. Its ability to balance esthetics, function, and patient comfort makes it a valuable tool in modern clinical practice.

### 4.7. Patients’ Comfort with Immediate Implant Therapy

Patients have consistently reported high comfort levels with immediate implants. This approach, involving the immediate placement and loading of implants, has been validated by extensive research as a method to significantly reduce overall treatment time and minimize the number of surgical interventions. These factors contribute to a quicker return to normal function and everyday activities, enhancing the patient experience and satisfaction [60]. Randomized controlled trials and longitudinal clinical follow-up studies provide robust evidence that immediate implant placement and provisionalization lead to marked improvements in several domains of patient comfort. Specifically, measures such as overall comfort, eating comfort, speaking comfort, and perceived esthetics show significant enhancement within the first week after the procedure, offering patients noticeable benefits early in their recovery [61,62]. Furthermore, immediate implant procedures have been associated with notable improvements in quality of life and overall patient satisfaction. These enhancements stem from the streamlined treatment process and the rapid restoration of both functional and esthetic aspects of oral health. Patients often express a high level of enthusiasm for the procedure, citing its convenience and efficiency. This positive feedback reflects the impact of immediate implants on their ability to resume daily activities and social interactions with restored confidence in appearance and functionality. Consequently, patients frequently recommend this treatment to others, underscoring its effectiveness in meeting their needs and expectations.

### 4.8. Managing Complications

Complications such as fenestration and dehiscence are well-documented in the literature regarding immediate implant therapy. The maxillary anterior and premolar regions are frequently cited as the most common sites for these issues. Fenestration typically occurs during the initial osteotomy phase, often due to over-orientation of the osteotomy towards the facial aspect. To address this, delayed implant placement is recommended for optimal bone healing and implant stability [63,64]. In cases of dehiscence, the placement of a resorbable or non-resorbable membrane, with or without the addition of bone particles, is advised to promote guided bone regeneration and support soft tissue healing [65].

Surgical trauma is another potential complication, commonly caused by overheating the bone during osteotomy. This trauma can lead to necrosis of the surrounding bone tissue, jeopardizing implant osseointegration. However, such outcomes can be mitigated by using copious irrigation during drilling, routinely replacing twist drills to maintain sharpness, and strictly adhering to the speed and pressure guidelines provided by the implant manufacturer [66].

Infections represent a significant risk factor for implant failure. To reduce this risk, premedication with broad-spectrum antibiotics is recommended, along with the implementation of a strict antiseptic protocol during surgery. Thorough debridement of any residual infection in the extraction socket is critical. In cases of active diffuse infection, it is generally advised to delay implant placement to ensure the infection is fully resolved before proceeding [67]. Implant malposition occurs when a suboptimal restorative-driven position is planned or executed. Minor positional issues may be corrected using prosthetic components, such as angulated abutments or cemented crowns. However, in cases of significant malposition, explantation followed by the placement of a new implant in the correct position may be necessary to achieve the desired functional and esthetic outcomes [68,69].

### 4.9. Limitations

This narrative review has certain limitations that should be acknowledged. First, the literature search was conducted using a limited number of databases, specifically PubMed and Google Scholar. While these platforms provide a substantial amount of information, the reliance on only these sources may have excluded relevant studies from other databases. Additionally, the search was restricted to articles published in English, potentially overlooking valuable research published in other languages. The search strategy used was narrowly focused and did not follow the rigorous framework of the Preferred Reporting Items for Systematic Reviews and Meta-Analyses (PRISMA) guidelines, which are designed to enhance the transparency and reproducibility of systematic reviews. The accompanying case illustration also has notable limitations. The results are based solely on qualitative analysis, primarily relying on the patient’s satisfaction with the esthetic and functional outcomes. Quantitative data, such as objective measurements of soft tissue stability, bone remodeling, or implant integration, were not included. Furthermore, the case study features only a short-term follow-up period, which limits the ability to evaluate the long-term success and stability of the treatment. Future clinical reports should aim to address these limitations by incorporating longer follow-up periods, objective quantitative assessments, and comparisons of different ceramic materials for final implant-supported prostheses to enhance the understanding of optimal treatment approaches.

## 5. Conclusions

Immediate implant loading has become an acceptable and consistent treatment approach, offering positive survival rates, good esthetic results, and strong patient satisfaction. Advanced technologies, such as 3D CBCT planning and the use of surgical guides, play a crucial role in improving the precision, safety, and long-term success of implant placement. Moreover, combining immediate implants with bone grafting materials may effectively reduce post-extraction bone loss and support soft tissue stability, enhancing both functional and esthetic outcomes. Immediate provisionalization further reduces the overall treatment time while helping to preserve peri-implant soft tissue architecture, improve esthetics, and increase patient comfort. Altogether, these strategies reflect a comprehensive, patient-focused approach to modern implant therapy, delivering efficient, esthetically pleasing, and clinically successful outcomes when paired with careful case selection and adherence to evidence-based protocols.

## Figures and Tables

**Table 1:** Inclusion and exclusion criteria of the search performed.

Criterion	Inclusion	Exclusion
Time period	Publications available between January 2010 and March 2025	All publications published before January 2010
Language	English	Non-English.
Type of articles	All research types, including case studies (e.g., case reports, case series, clinical techniques) and literature reviews (e.g., systematic reviews and meta-analysis. Full text available.	Letters, books, book chapters, and full text not available

**Table 2:** This table summarizes case reports of immediate implants in the esthetic zone.

Authors/Year	Title	Methods	Results
Rathi M et al. 2025 [27]	Management of Single Tooth Replacement in the Esthetic Region Using Immediate Implant Placement: A Case Report	An implant (4.0 mm diameter) replacing a fractured maxillary right central incisor. The implant was placed in conjunction with a mixture of autologous and xenograft with collagen membrane.	Understanding hard and soft tissue anatomy, microesthetics, and patient expectations is imperative for evidence-based treatment planning and successful implant therapy.
Nguyen QV et al. 2023 [28].	Anatomically Driven Immediate Implant Placement in the Esthetic Zone: Two Case Reports as Proof of Principle	An implant (3.5 × 11.5 mm) replacing a fractured maxillary lateral incisor. Osteotomy guided by reinserted extracted root; implant placed flapless with sticky bone graft (PRF + cortical allograft).	Anatomical guidance via reinsertion of the root optimizes implant positioning along the palatal wall, enabling ideal 3D orientation and preserving soft tissue. The technique is effective, but the stability of the reinserted root may be challenging.
Yang C et al. 2021 [29].	Immediate Implant Placement and Provisionalization in the Esthetic Zone Using Flapless Technique	An implant (4.3 mm × 18 mm) replaced the maxillary right central incisor due to trauma.	Analyzing the CBCT for bone thickness and bone levels, as well as classifying the predicted extraction socket type, is important in choosing the correct surgical technique, graft materials, and membranes if needed; the type and size of dental implant; and making the decision to immediately load a provisional prosthesis.
Tarnow DP et al. 2017 [30].	Clinical Management of Type 3 Recession Defects With Immediate Implant and Provisional Restoration Therapy: A Case Report	Replacement of a maxillary right central incisor with internal root resorption and type 3 recession defect with an immediate implant combined with bone and soft tissue graft.	The use of immediate implant and provisional restoration therapy in type 3 (recession) clinical situations can result in predictable esthetic outcomes. The diagnostic keys for success are (1) pre-existing labial tooth malposition; (2) flapless tooth removal with the palatal tissues at the proper height; (3) palatal implant placement; (4) dual-zone bone grafting; (5) provisional restoration placement in non-occlusal function; and (6) proper tissue healing for 4 to 6 months.
Chandra Sekar A et al. 2012 [31]	Immediate Implant Placement: A Case Report	Replacement of maxillary lateral incisor due to trauma with an implant (3.7 mm × 13 mm) in conjunction with bone graft.	Aiming to reduce the process of alveolar bone resorption and treatment time, the immediate placement of endosseous implants into extraction sockets is known to achieve a high success rate of between 94 and 100%, compared to the delayed placement.
Fang J et al. 2021 [32]	Immediate implant placement in combination with platelet-rich fibrin into extraction sites with periapical infection in the esthetic zone: A case report and review of literature	An implant (3.3 × 12 mm) replacing a fractured maxillary central incisor with periapical infection. PRF mixed with xenograft (Bio-Oss) and layered with PRF-collagen-PRF membrane barrier.	PRF promoted soft/hard tissue healing and managed infection effectively, resulting in stable bone and esthetic outcomes at 1-year follow-up despite initial infection and bone defects.
Takvani R et al. 2024 [33]	Immediate Loading and Implant Placement with Bone Grafting in Severely Proclined Anterior Mobile Teeth in the Esthetic Zone: A Report of an Intriguing Case	Three implants (3.8 × 13 mm and 3.75 × 10 mm) replaced four upper anterior teeth. Implants were placed using a surgical guide and flapless extraction. Bone graft included autograft and xenograft (Bio-Oss).	Immediate implant placement with grafting and provisional bridge achieved primary stability (35–40 Ncm). At 1-year follow-up, stable soft tissue contour and healthy marginal bone levels were observed.
Fernandes G et al. 2024 [34]	Customizing the Emergence Profile Around an Immediately Loaded Single Implant in the Esthetic Zone: A Case Report	An implant (4 × 11.5 mm) was placed in the maxillary central incisor region after ridge augmentation with autogenous block graft and xenograft. A customized emergence profile was developed using composite interim restoration.	The emergence profile was shaped incrementally over 2 months. Final restoration transferred tissue contours accurately using a custom impression coping. Esthetic results were harmonious with adjacent teeth.
Furtado Araújo MV et al. 2015 [35]	Immediate Placement and Restoration of an Implant in an Infected Socket in the Esthetic Zone	An implant (4.1 × 12 mm) replacing a fractured maxillary central incisor with periapical lesion. Atraumatic extraction, xenograft in buccal gap, and immediate screw-retained provisional crown.	At 12-month follow-up, facial soft tissue and hard tissue contours were symmetrical and stable. Highlights the importance of biotype, implant depth, and prosthetic emergence profile.
Jundaeng J et al. 2025 [36]	Dental implant in esthetic zone: A case report	An implant (3.5 × 10 mm) was placed in the upper left central incisor region with labial thread exposure. Papilla preservation flap and xenograft bone graft were used to manage concavity. Healing abutment placed for non-submerged healing.	Four-month osseointegration followed by E-max CAD crown on anodized abutment. At the 3-month follow-up, the esthetic outcome was natural, with stable gingiva and no bone loss.

**Table 3:** This table summarizes systematic reviews related to immediate implant therapy.

Authors/Year	Title	Methods	Results
Chen R et al. (2025) [37]	Effectiveness of Immediate Implant Placement into Defective Sockets in the Esthetic Zone: A Systematic Review and Meta-analysis	A systematic review and single-arm meta-analysis of 23 clinical studies from 2000 to 2022 evaluating 630 implants placed in sockets with defects in the anterior esthetic region. Outcomes assessed included survival rate, marginal bone loss (MBL), gingival recession, and pink esthetic score (PES).	Immediate implant placement (IIP) into defective sockets showed a 98.1% survival rate. MBL was 1.03 mm at 6 months, 0.72 mm at 12 months, and 1.15 mm at ≥24 months. Gingival recession at 12 months averaged 0.25 mm. PES at 12 and ≥24 months were 12.34 and 12.58, respectively. IIP in socket defects is feasible with favorable outcomes.
Hamilton A et al. (2023) [38]	Selection criteria for immediate implant placement and immediate loading for single tooth replacement in the maxillary esthetic zone: A systematic review and meta-analysis	Systematic review and meta-analysis of 68 studies evaluating Type 1A protocols (immediate implant placement and immediate loading). Inclusion: single implants (15–25 FDI) with a minimum 12-month follow-up. Risk assessments conducted per site-specific factors.	Type 1A protocol shows high survival (97.7%) and early survival (98.3%) rates. Facial bone gap > 2 mm and presence of chronic endodontic infection were associated with higher success. Strict selection criteria are crucial for predictable outcomes.
Qin R el al (2023) [39]	Immediate Implant Placement with or Without Immediate Provisionalization in the Maxillary Esthetic Zone: A Systematic Review and Meta-analysis	Systematic review of 8 RCTs comparing immediate implant placement with immediate loading (IPIL) vs. delayed loading (IPDL) in the maxillary esthetic zone. Outcomes included midfacial mucosal level, papilla height, marginal bone loss, survival, and peri-implant soft tissue health.	IPIL showed significantly less midfacial mucosal recession (0.48 mm difference), better papilla preservation (SMD –0.16), and improved ridge dimension (SMD 0.94). No significant differences were found in implant survival, bone loss, probing depth, or plaque score.
Seyssens L. et al. (2020) [40]	Immediate implant placement with or without connective tissue graft: A systematic review and meta-analysis	Systematic review and meta-analysis of 5 RCTs and 3 non-RCTs (409 implants). Compared immediate implant placement (IIP) with vs. without buccal connective tissue graft (CTG). Primary outcome: vertical mid-facial soft tissue changes. Minimum 12-month follow-up.	CTG significantly improved vertical mid-facial tissue stability (mean gain: 0.41 mm; *p* < 0.001) and reduced risk of ≥1 mm soft tissue asymmetry 12-fold. No significant differences in pink esthetic score, marginal bone level, or probing depth. CTG recommended when high risk of recession exists.
Wittneben JG et al. (2023) [41]	Clinical performance of immediately placed and immediately loaded single implants in the esthetic zone: A systematic review and meta-analysis	Systematic review and meta-analysis of 63 studies (10 RCTs, 28 prospective, 25 retrospective) on Type 1A implant placement (immediate placement and loading) in the maxillary esthetic zone. Primary outcomes: implant and prosthetic survival. Secondary: PES, WES, midfacial recession, papilla height.	Implant survival: 99.2% at 1 year, 97.5% at 3 years, 95.8% at 5 years. Restoration survival: 98.9% at 1 year, 96.8% at 2 years, 94.8% at 5 years. Mean PES gain: +0.82. Papilla height showed a decrease (–0.71 mm), and midfacial recession was minimal (–0.15 mm).
Velasco Bohórquez P el al (2021) [42]	Failure Rate, Marginal Bone Loss, and Pink Esthetic with Socket-Shield Technique for Immediate Dental Implant Placement in the Esthetic Zone. A Systematic Review and Meta-Analysis	Systematic review and meta-analysis of 16 studies (RCTs, prospective, retrospective, case series). Compared socket-shield technique (SST) to conventional immediate implant placement (CIIP) for failure rate, marginal bone loss, and pink esthetic score (PES).	SST showed a 1.37% implant failure rate (CI: 0.21–2.54%)—no difference vs. CIIP. SST had significantly less marginal bone loss (–0.5 mm, *p* < 0.01) and higher PES (mean difference: 1.15 points). PES improved with time (0.02 points/month, *p* = 0.049). Recommended for preserving esthetics.
Slagter KW et al.(2014) [43]	Immediate placement of dental implants in the esthetic zone: a systematic review and pooled analysis	Systematic review of 34 studies (RCTs, prospective, and retrospective) evaluating immediately placed single implants in the esthetic zone. Pooled analysis examined implant survival, marginal bone loss (MBL), and interproximal and midfacial tissue levels.	Implant survival was 97.1% (95% CI: 95.8–98.0). Mean MBL: 0.81 ± 0.48 mm. Interproximal mucosal loss: 0.38 ± 0.23 mm; midfacial mucosal loss: 0.54 ± 0.39 mm. Delayed provisionalization, flap use, and CT grafts were associated with >0.5 mm MBL. Immediate provisionalization may favor stability.
Mao Z el al (2021) [44]	Buccal bone dimensional changes at immediate implant sites in the maxillary esthetic zone within a 4-12-month follow-up period: A systematic review and meta-analysis	Systematic review and meta-analysis of 16 studies (4 RCTs, 12 NRCTs; 568 implants) evaluating horizontal (CHBD) and vertical (CVBD) buccal bone changes 4–12 months after immediate implant placement in the maxillary esthetic zone. Meta-regression analyzed the effect of grafting, flap design, and restoration protocol.	CHBD: 0.71 mm, CVBD: 0.58 mm. Bone grafting significantly reduced CHBD (mean reduction 0.46 mm, *p* = 0.015). Flapless approach and immediate provisionalization were associated with less resorption, though not statistically significant. Immediate placement does not prevent buccal bone loss.
Chen H et al.(2018) [45]	Immediate placement of dental implants into infected versus noninfected sites in the esthetic zone: A systematic review and meta-analysis	Systematic review and meta-analysis of 9 clinical studies (n = 1735 implants) comparing immediate implant placement into infected vs. noninfected extraction sockets in the esthetic zone. Outcomes included implant survival, bone level, and gingival level changes.	Similar implant survival rates in infected (97.6%) and noninfected (98.4%) sites (RR = 0.99; *p* = 0.138). No significant differences in bone level change (MD = 0.03 mm) or gingival level change (MD = –0.06 mm). Thorough debridement remains essential for success.
Cheng Q et al.(2020) [46]	Clinical Outcomes Following Immediate Loading of Single-Tooth Implants in the Esthetic Zone: A Systematic Review and Meta-Analysis	Systematic review and meta-analysis of 7 RCTs (n = 386) evaluating single-tooth immediate implants. Most studies used a parallel design; one multicenter study included both parallel and split-mouth designs. Immediate loading (within 24 h) was compared to conventional loading, assessing implant survival, marginal bone loss, soft tissue changes, esthetic outcomes, and patient satisfaction.	No significant difference in 1-year implant survival (RR = 0.99; 95% CI: 0.95–1.02). MBL and soft tissue changes were comparable. Esthetic outcomes and patient satisfaction were similar between immediate and conventional loading protocols.

## Data Availability

Data are contained within the article.
